# Cross-Cultural Studies Into Gambling Consumption Behavior: Eyeing Eye-Tracking Measures

**DOI:** 10.3389/fpsyg.2021.646007

**Published:** 2021-06-10

**Authors:** En Li, Donnel A. Briley, Mike J. Dixon, Robert J. Williams

**Affiliations:** ^1^School of Business and Law, Central Queensland University, Rockhampton, QLD, Australia; ^2^The University of Sydney Business School, University of Sydney, Sydney, NSW, Australia; ^3^Department of Psychology, University of Waterloo, Waterloo, ON, Canada; ^4^Faculty of Health Sciences, University of Lethbridge, Lethbridge, AB, Canada

**Keywords:** culture, cross-cultural, gambling, gambling consumption behavior, visual attention, eye-tracking

## Introduction

“Every man's ability may be strengthened or increased by culture”—John Abbot (Textappeal, 2017).

While data aggregated from gambling operators have shown cross-cultural differences in the behavior of their customers (CasinoBeats, 2020), in recent years research into gambling consumption has been strengthened and enriched by studies uncovering the roles of culture in shaping gambling relevant phenomena (Oei et al., 2019). These studies were commonly based on data collected through survey questionnaires (e.g., Rinker et al., 2016; Calado et al., 2020) or interviews (e.g., Radermacher et al., 2016; Egerer and Marionneau, 2019). In the present opinion paper, it is argued that eye-tracking measurements should also be adopted in cross-cultural gambling research, particularly given the systematic differences in visual attentional patterns that potentially exist among gambling product consumers from different cultures.

## Visual Attentional Patterns: Cross-Cultural Differences

A main focus of psychological research spanning the last few decades, has been cultural differences in human behaviors (e.g., in information processing and associated decisions/judgments, Briley et al., 2014). Specifically, research findings have suggested the following cross-cultural differences in visual attentional patterns: westerners are more likely to allocate their visual attention less broadly and more to focal/salient objects independently of their contexts compared to easterners, who are more likely to attend more to contextual information as well as the relationships between the objects and their contexts (e.g., Kitayama et al., 2003; Chua et al., 2005; Masuda and Nisbett, 2006; Boduroglu et al., 2009; Zhang and Seo, 2015). For example, Chua et al. (2005) examined the eye movements of both European American and Chinese participants during their viewing of pictures that included a single foregrounded object with realistic background on each of them (e.g., a picture showing a plane with clouds, sky, and mountains in the background). Chua et al. (2005) found that European Americans spent more fixation time on the foregrounded objects, and looked at those objects more quickly, as compared to their Chinese counterparts, who on the other hand, demonstrated higher number of fixations to the background than did European Americans. Chua et al. (2005) also found that Chinese participants' ability to recognize previously seen foregrounded objects dropped when these objects were presented with new rather than old backgrounds, confirming that easterners tend to tie objects with backgrounds in their perceptual processes. In addition, Boduroglu et al. (2009) compared Americans with East Asians on their capability to detect visual changes. They discovered that Americans were more capable than East Asians to detect color changes when a layout of colored squares was shrunk to a smaller region, and less capable of doing so when the layout was expanded to a larger region (Boduroglu et al., 2009). Moreover, Americans were quicker than East Asians in detecting visual changes that appeared at the center of the screen (Boduroglu et al., 2009). In a study reported by Kitayama et al. (2003), Japanese participants and European American participants completed a series of absolute or relative line drawing tasks. In each of those tasks (Kitayama et al., 2003), participants were presented an original line printed within an original square, and were required to draw a new line within a new square, identical to the original line in either the absolute length (e.g., 62 mm; absolute line drawing task) or the relative length compared to the square in the context (e.g., 70% of the contextual square's height; relative line drawing task). Kitayama et al. (2003) discovered that the Japanese participants performed more accurately in the relative line drawing tasks (suggesting their better ability to incorporate the contextual information), and the European American participants performed more accurately in the absolute line drawing tasks (suggesting their better ability to ignore the squares in the context). In two of the experiments documented in Masuda and Nisbett (2006), Japanese participants and American participants were presented with animated vignettes which had changes in the contextual information as well as the focal object information. In those experiments, the Japanese performed better than the Americans in detecting the contextual changes, whereas the Americans performed marginally better or better than the Japanese in detecting changes in the focal object information (Masuda and Nisbett, 2006). Zhang and Seo (2015) tracked American and Chinese participants' eye movements when they viewed pictures that included food items with varying degrees of background saliency (without the presence of any other particular task). They found the Americans looked at the food items faster than the Chinese (namely, the Americans were less distracted by the background context, Zhang and Seo, 2015).

## Gambling Consumption Behavior and Eye-tracking Measures: An Exemplar Phenomenon

Attentional measures including direct (e.g., eye movement based) and indirect measures (e.g., reaction time based) have been featured in gambling research (e.g., Brevers et al., 2011; Grant and Bowling, 2015). But to the best of our knowledge eye-tracking measurements still have not been adopted in cross-cultural gambling research, despite the existence of cross-cultural differences in visual attentional patterns, as described in the previous section. Next, we will propose an exemplar gambling consumption phenomenon that is potentially linked to those cross-cultural differences and can be examined and better understood by tracking the eye movements of consumers while engaged in the target activity.

This phenomenon is based on the immersion (Murch et al., 2020) or dark flow state (Dixon et al., 2018) consumers may experience during their engagement with electronic gaming machines (EGMs). The experience of this state may occur when consumers are highly focused on their EGM play while losing/reducing attention paid to stimuli other than the EGM they are playing, and this experience can be measured by items such as “I felt completely absorbed” (Murch et al., 2020, p. 1129), “I was fully occupied with the game” (Dixon et al., 2018, p. 78), and “I lost connection with the outside world” (Dixon et al., 2018, p. 78). The dark flow state is an important gambling consumption phenomenon, since if players' attention is wholly immersed in they may find themselves spending more time and money than intended. Indeed, dark flow has repeatedly been shown to be correlated with the severity of gambling problems (Dixon et al., 2018). Moreover, immersion reported by non-problem EGM gamblers who played on an authentic EGM was associated positively with the ratio of dwell time to the EGM's credit window against the EGM's reels (Murch et al., 2020; note that Murch and his colleagues used the term dwell time to represent “the percentage of task time spent fixating on a given area-of-interest,” p. 1129). Two opposite predictions could be proposed and tested regarding this phenomenon. On one hand, as reels are typically located on the center of EGM screens and credit windows commonly appear close to the edge of EGM screens, easterners, as compared to westerners, could allocate more visual attention to the credit windows when playing EGMs, or display higher ratios between their dwell time to credit windows against reels. On the other hand, eastern consumers could also be more likely than their western counterparts to visually attend to the contextual information outside the screen of the EGM they are playing, be more likely to get visually distracted by off-screen stimuli in the background, and be less likely to lose connections to the outside world. Hence, the first prediction points to greater amount of EGM immersion being experienced by average eastern consumers, whereas the second prediction suggests that average western consumers could be more immersed when playing EGMs. These potential cross-cultural differences in EGM immersion can be tested through eye-tracking measures such as the proportion of EGM playing time spent in fixating on the EGM reels, the EGM credit window, and any stimuli outside the EGM screen, and the amount of saccades that cross the edge of the EGM screen. Even though researchers have, in the past, explored and found cross-cultural variations in certain flow phenomena that were of little direct gambling relevance (e.g., Moneta, 2004; Liu et al., 2015), as far as we are aware no studies have focused on the potential cross-cultural differences in EGM immersion/flow experiences. Applying eye-tracking measures to the examination of EGM immersion among both western and eastern consumers, should directly address this gap in the literature.

In addition to their roles in testing the potential cross-cultural differences in EGM immersion, eye-tracking measures can be further utilized to identify effective strategies to prevent or reduce the negative consumer consequences due to EGM immersion (e.g., harms caused by excessive EMG gambling). Specifically, these strategies could be centered around suitable responsible gambling pop-up messaging being designed and implemented to the EGM screens, since pop-up messaging could potentially capture EGM players' visual attention, disrupt their EGM immersion, and provide them responsible-gambling-oriented information (e.g., clues or warnings about their time/monetary expenditure). In a recent systematic review and meta-analysis study, Bjørseth et al. (2021, p. 15) stated that “As gambling becomes increasingly digitalized, pop-up messaging appears to represent an accessible and cost-effective way to attenuate excessive gambling behavior and to modify gambling-related cognitions.” However, we propose that such pop-up messaging features should be differentially designed for EGMs targeting western versus eastern consumers, and that eye-tracking measures should be relied upon to determine the appropriate visual aspects (e.g., location, size) of these pop-up messages, for them to effectively attract the visual attention of their target consumers.

## Discussion

To date, research into gambling consumption behavior has covered a wide range of topic areas, such as Internet gambling (e.g., Williams et al., 2012), EGM gambling (e.g., Dixon et al., 2013; Li et al., 2016; Thorne et al., 2016), sports betting (e.g., Hing et al., 2018; Li et al., 2020), and gambling harms (e.g., Li et al., 2017; Jeffrey et al., 2019). The present opinion paper attempts to further advance this line of research, by highlighting the potential value of eye-tracking measures being adopted in cross-cultural research on gambling consumption behavior. As an exemplar phenomenon, it is proposed that consumers from western and eastern cultures could be differentiated in terms of their susceptibility to EGM immersion, and that the attentional mechanisms driving the corresponding cross-cultural differences could be pinpointed through eye-tracking measures. Moreover, eye-tracking measures could be further relied upon in designing culturally effective pop-up messages to prevent or reduce the negative impacts of EGM immersion. In addition to the proposed exemplar phenomenon, eye-tracking measures could also be utilized in addressing other research questions linked to potential cross-cultural differences in gambling consumption. For example, in lottery consumption, do average western and eastern consumers pay different attention to the numbers located around the center or the edge of the lottery tickets; and if such attentional differences exist, do they affect the likelihood of those numbers being picked? In addition, advertisements for gambling products can include responsible gambling messages for the purpose of minimizing or preventing harms caused by the advertised gambling products (e.g., “Is gambling a problem for you? Call Gambling Help on 1800 858 858 or visit “gamblinghelponline.org.au”, Lole et al., 2019, p. 501). Hence, when exposed to gambling advertisements, are average western or eastern viewers more likely to notice the responsible gambling messages if such messages are presented around the center or the edge of the advertisements? And if cross-cultural differences indeed arise in the likelihood of responsible gambling messages being noticed, can they also have impacts on the amount of gambling harms experienced by consumers from different cultures? Examining the proposed exemplar phenomenon as well as these further questions cannot only advance the psychological theories associated with gambling consumption behavior, these research efforts might also shape policies, regulations, or practice associated with promoting responsible gambling as well as reducing or preventing gambling harms in different cultures.

## Author Contributions

EL made significant contributions to this manuscript. All authors contributed to the article and approved the submitted version.

## Conflict of Interest

The authors declare that the research was conducted in the absence of any commercial or financial relationships that could be construed as a potential conflict of interest.
